# Chirurgische Nadeln in Orthopädie und Unfallchirurgie

**DOI:** 10.1007/s00064-021-00734-7

**Published:** 2021-09-16

**Authors:** Klaus Dresing, Martin Franz Langer, Theddy Slongo

**Affiliations:** 1 Klinik für Unfallchirurgie, Orthopädie und Plastische Chirurgie, Universitätsmedizin Göttingen, Robert-Koch-Str. 40, 37075 Göttingen, Deutschland; 2 Klinik für Unfall‑, Hand- und Wiederherstellungschirurgie, Universitätsklinikum Münster, Waldeyerstr. 1, 48149 Münster, Deutschland; 3 Paediatric Trauma and Orthopaedics, University Children’s Hospital, 3010 Berne, Schweiz

**Keywords:** Nadelform, Konische Nadeln, Stumpfe Nadeln, Schneidende Nadeln, Nadelhalter, Needle shape, Conical needle, Blunt needle, Cutting needle, Needle holder

## Abstract

Für Chirurgen in der Klinik und Praxis ist der Umgang mit Nahtmaterial das „tägliche Brot“. Daher könnte man annehmen, dass die Kenntnisse dieses Materials kompetent und umfassend sind. Die tägliche Erfahrung zeigt indes ein anderes Bild. Oft sind die Kenntnisse bezüglich Nadelform, Nadelbeschaffenheit sowie Nadelgröße nur marginal und der Chirurg muss sich auf die Kenntnisse seiner OP-Pflegefachkraft verlassen. Die Wahl der Nadel ist für jeden Operationsschritt und jedes Gewebe ausschlaggebend. Die Verwendung des korrekten Nahtmaterials in Bezug auf das zu nähende Gewebe ist jedoch essenziell, einerseits hinsichtlich der Qualität der Naht, aber auch hinsichtlich der Traumatisierung des Gewebes andererseits. Je nach Gewebe sind an Nadel wie auch an Fäden verschiedene Anforderungen zu stellen respektive zu berücksichtigen. Mit diesem Beitrag sollen die wesentlichsten und grundlegendsten Kenntnisse vermittelt werden wie Einfluss der Nadelform und Dimension, Auswirkung von atraumatischen respektive traumatischen (sog. scharfe) Nadeln auf das Gewebe, die Form der Nadelspitze. Das Zusammenspiel zwischen Nadelhalter und Nadel und der Hand des Operateurs bei verschiedenen Anwendungen wird dargestellt. In diesem Beitrag wird das Hauptaugenmerk auf Nadel und Nadelhalter gelegt. In Planung ist ein Nachfolgebeitrag, der sich speziell dem Nahtmaterial widmet. Der Beitrag erhebt nicht den Anspruch auf 100 %ige Vollständigkeit, es sollen jedoch die wesentlichsten, alltäglich vorkommenden Fragen geklärt werden.

**Zusatzmaterial online:**

Die Online-Version dieses Beitrags (10.1007/s00064-021-00734-7) enthält eine Vergleichstabelle der chirurgischen Nadeln verschiedener Hersteller (ESM2) inklusive Erläuterungen (ESM1) sowie 2 Videos zu den Nadelhaltergriffen: Video 1 Nadelhaltergriff: Daumen-Ringfinger-Griff (ESM3) und Video 2 Nadelhaltergriff: Daumenballengriff (ESM4) (Videos: Mit freundl. Genehmigung von © Dr. Theddy Slongo [Autor der Videos], Inselspital Bern, Paediatric Trauma and Orthopaedics, University Children’s Hospital, Dept. of Paediatric Surgery. Alle Rechte vorbehalten).

## Abstract (en)

For surgeons in the clinic and practice, the handling of suture material is the “daily bread”. Therefore, one might assume that knowledge of this material is competent and comprehensive. However, daily experience shows a different picture. Often, the knowledge regarding needle shape, needle composition as well as needle size is only marginal and the surgeon has to rely on the knowledge of his OR nurse.

The choice of needle is critical for each surgical step and each tissue; however, the use of the correct suture material in relation to the tissue to be sutured is essential, not only with respect to the quality of the suture but also with respect to the traumatization of the tissue. Depending on the tissue, different requirements must be placed on or taken into account for both the needle and the sutures. The purpose of this article is to provide the most essential and basic knowledge, such as the influence of needle shape and dimensions, effect of atraumatic or traumatic (so called sharp) needles on the tissue and shape of the needle tip. The interaction between the needle holder and the needle and the surgeon’s hand in different applications is presented. In this present article, the main focus is on the needle and needle holder. A follow-up article specifically dedicated to sutures is being planned. The article does not claim to be 100% complete but the most essential, everyday questions should be clarified.

## Lernziele

Nach Lektüre dieses Beitrags …

- haben Sie eine Basis für ihre chirurgische Tätigkeit zur „optimalen Naht“,
- treffen Sie die Entscheidung bezüglich Nahtmaterial selbst und überlassen dies nicht wie so oft dem Personal,
- kennen Sie die chirurgischen Nadeln, deren Eigenschaften sowie Vor- und Nachteile.

## Einleitung

Die Nadeln von chirurgischem Nahtmaterial sind ein entscheidender Faktor beim Wundverschluss. Die **chirurgische Nadel**

chirurgische Nadel

platziert und führt das Nahtmaterial ins Gewebe. Für die optimale **chirurgische Naht**

chirurgische Naht

stellt neben dem Operateur selbst das chirurgische **Nahtmaterial**

Nahtmaterial,

also Nadel und Faden, einen entscheidenden Faktor dar. Dabei wird das Nahtmaterial von der jeweiligen, für ein bestimmtes Gewebe vorgesehenen Nadel möglichst atraumatisch durchs Gewebe geführt. Dabei muss sie in der Lage sein, das Nahtmaterial mit kleinstem Trauma durch das Gewebe zu führen. Zu oft wird jedoch nicht dem Gewebe angepasstes Nahtmaterial verwendet, was dann zu Nahtproblemen führen kann. Dass dem so ist, ist nicht nur auf mangelnde Kenntnis des chirurgischen Nahtmateriales durch den Chirurgen zuzuführen, sondern auch der weltweit nahezu unübersichtlichen Menge an verschiedenen Nadeltypen, Nadelformen und Fäden, die dann noch nach verschiedenen Normen geregelt sind, zuzuschreiben. Somit hängt die richtige Wahl von Nadel und Faden von dem zu fixierenden Gewebe, respektive dem entsprechenden Operationsgebiet ab.

Da es sich bei der Aufarbeitung dieses Beitrag gezeigt hat, dass die Menge an Nadeln und Fäden sowie auch deren Nomenklatur dermaßen unterschiedlich und von der Menge her sehr groß sind, sollen hier die in Zentraleuropa meistverwendeten Produkte beschrieben werden.

Die **Nadelkonfiguration**

Nadelkonfiguration

und die adäquate **Auswahl der Nadel**

Auswahl der Nadel

sind für das operative Verfahren ausschlaggebend. Die vielen Konfigurationen von chirurgischen Nadeln und die Wahl der geeigneten Nadel durch den Chirurgen hängen ab von der Art der Wunde oder des Gewebes, das operiert wird und genäht werden soll, und damit von dem Operationsgebiet und dem richtigen Verhältnis zur Größe des Nahtmaterials. Die chirurgische Nadel sollte dabei in der Lage sein muss, das Nahtmaterial mit **minimalem Trauma**

minimalem Trauma

durch das Gewebe zu führen.

Sämtliche Aspekte dieses chirurgischen Werkzeugs können nicht abgehandelt werden. Es sollen grundlegende Kenntnisse von chirurgischen Nadeln erörtert werden, wobei der Schwerpunkt auf Nadeln liegt, die in Orthopädie und Unfallchirurgie Anwendung finden.

## Nadelanforderungen

Der Chirurg wünscht sich natürlicherweise für jede Operationssituation unabhängig von Gewebe und der anatomischen Situation ein „universales“ Nahtmaterial, mit dem er ohne Wechsel des Materials alles nähen kann. Wie wir jedoch wissen, existiert ein solches Produkt nicht, da jede Körperregion, jedes Gewebe und jede Situation **individuelle Anforderungen**

individuelle Anforderungen

an die Naht stellen und somit entsprechendes Nahtmaterial benötigen. Wie die Gewebeeigenschaften in verschiedenen Regionen des Körpers oder Schichten unterschiedlich sind, so sind auch die Anforderungen an das Nahtmaterial, hier die Nadeln, unterschiedlich. Die nachfolgend aufgeführten Eigenschaften sind für chirurgische Nadeln zu unterscheiden.

### Nadelschärfe

Eine optimale Nadel sollte das Gewebe mit **geringem Widerstand**

geringem Widerstand

/Druck durchdringen können, ohne dabei ein Gewebetrauma größeren Ausmaßes zu hinterlassen. Es darf bemerkt werden, dass das Durchführen der Nadel durch das Gewebe durch den Chirurgen mindestens von identischer Bedeutung ist.

Die Nadel muss scharf genug sein, um Gewebe mit geringstem Widerstand zu penetrieren, damit ein leichtes und schnelles Eindringen in das Gewebe ermöglicht wird.

### Durchzugverhalten

Beim Eindringen in und durch Haut und Gewebe sollten Nadel und Faden im Durchzugkanal zu wenig zusätzlicher Schädigung führen. Eine glatte und besonders eine beschichtete Nadeloberfläche erleichtern dies. Der Durchmesser sollte möglichst klein sein. Auch das Herausziehen der Nadel muss ohne zusätzliches Trauma möglich sein.

### Biegefestigkeit

Darunter verstehen wir den Wert, wie leicht sich eine Nadel verbiegen lässt. Prinzipiell sollte eine Nadel 2 Eigenschaften aufweisen: einerseits so steif respektive biegefest sein, dass sie sich nicht verformt, und andererseits doch eine gewisse Elastizität aufweisen. Wir nennen dies **stabil-elastisch**

stabil-elastisch.

Die Nadel sollte so steif, bzw. biegefest sein, dass sie sich bei der Naht nicht verformt. Die Biegefestigkeit gibt an wie leicht sich die jeweilige Nadel verbiegen lässt.

### Elastizität

Das Nadelmaterial sollte so elastisch und flexibel sein, dass **Nadelbrüche**

Nadelbrüche

möglichst vermieden werden. Die Nadel sollte also eine gewisse Elastizität aufweisen, sodass sie nicht permanent verbogen bleibt und bei gewissem Druck wieder in die ursprüngliche Form zurück „federt“. Dadurch sollten auch Nadelbrüche verhindert werden.

### Größe

Die Nadel sollte so dünn, schlank sein, dass das Nadeltrauma möglichst gering bleibt.

### Korrosionsbeständigkeit

Das Nadelmaterial sollte **rostfrei**

rostfrei

und korrosionsbeständig sein. So können Reaktionen mit dem Gewebe oder Fremdkörperreaktionen vermindert werden.

### Sterilität

Prinzipiell wird heute in den meisten Ländern von allen Herstellern das gesamte Nahtmaterial, Nadel/Faden, steril verpackt der Klinik zur Verfügung gestellt. Diese Packungen sollte nicht beschädigt oder aufgebrochen werden und nicht einem hausinternen Sterilisationsprozess zugeführt werden. Dies kann neben der möglichen nicht optimalen Sterilisation die von der Firma garantierte Qualität beeinflussen! Eine Inokulation von Keimen in das Gewebe kann durch **steriles Nahtmaterial**

steriles Nahtmaterial

verhindert werden.

### Gute Griffigkeit für den Nadelhalter

Ein sicherer Griff der Nadel im Nadelhalter, sodass sie sich nicht im Halter drehen kann, führt die Nadel im gewünschten Winkel in das Gewebe, ohne während des Eindringvorgangs und des Durchtritts durch das Gewebe die **Position**

Position

zu ändern. Um bei den oben erwähnten Voraussetzungen die Naht schlussendlich perfekt ausführen zu können, sollte ein der Nadeldimension, Nadelkrümmung und Nadelhärte angepasster Nadelhalter verwendet werden. Voraussetzung für den Nadelhalter ist, dass er auf einer möglichst kleinen Strecke die Nadel perfekt festhält, ohne die Nadel zu traumatisieren und zu verkrümmen.

## Nadeleigenschaften

### Eindringkraft/Schärfe

Abhängig von der Form der Nadel und insbesondere der Spitzenkonfiguration ist der Einstechwiderstand abhängig von der Gewebeart und der Stabilität des Gewebes und selbstverständlich von der Konfiguration von Spitze und Design der Nadel. Bei plastisch-chirurgischen Eingriffen werden meist kleine scharfe Nadeln verwendet. Ein bekannter Spruch ist, je schärfer, desto weniger **Narbenbildung**

Narbenbildung

[1]. Die Gefahr zu scharfer Nadeln auf der anderen Seite soll nicht unerwähnt bleiben, da hierbei die Kontrolle der Nadelpassage durch das Gewebe verloren gehen kann.

### Elastizität

Elastizität ist die Fähigkeit des Materials, die Ausgangsform wieder einzunehmen. Beim Einstechen und der Kraftübertragung von der Hand des Operateurs über den Nadelhalter auf die Nadel werden Verformungskräfte auf das Material übertragen. Die Verformbarkeit, auch als Duktilität bezeichnet, gibt an, wann der **Elastizitätsmodul**

Elastizitätsmodul

überschritten ist und die Nadel bricht [2]. Dieser Faktor ist abhängig von den verwendeten Stahleigenschaften. Die Duktilität nimmt zu von 420er-Stahl zu 300er-Stahl. Normalerweise wird sich eine Nadel mit hoher Duktilität erst verbiegen, bevor sie bricht.

### Biegefestigkeit

Der kritischste Aspekt der Nadelfestigkeit ist die permanente Verbiegung der Nadel. Wichtig ist, wie viel **Winkelverformung**

Winkelverformung

die Nadel aushält, bevor sie sich dauerhaft verformt. Dieser Punkt liegt in der Regel zwischen 10 und 30°, abhängig vom Material und dem Herstellungsprozess [1]. Eine verbogene Nadel sollte der Operateur nur in seltenen Fällen wieder zurückbiegen.

### Biegekraft

Die Biegekraft gibt an, wie viel Kraft benötigt wird, die Nadelform zu verbiegen. Mit höherem **Radius**

**Figure 1:**
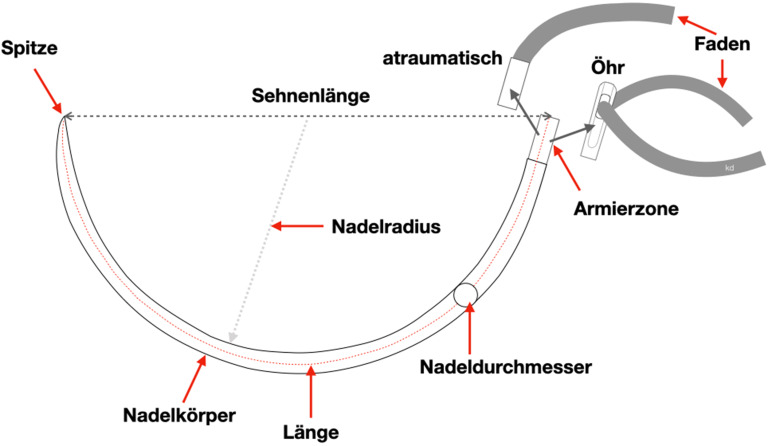

Radius

nimmt die Biegekraft zu, also z. B. von HR22 zu HR36 (Abb. 1 und 6). Eine schwache Nadel, die sich zu leicht verbiegt, beeinträchtigt die Kontrolle des Chirurgen über die Nadel und kann das umliegende Gewebe beschädigen [1].

#### Merke

Die Nadelbiegung/-krümmung kann durch die Breite der Nadelhalterbranchen verändert werden.

## Nadelmaterial

Nach AISI (American Iron and Steel Institute) werden die für Nadeln verwendete **Edelstähle**

Edelstähle

eingeteilt: 420er-Stahl, 455er-Stahl und 300er-Stahl. Stahl der 420er-Sorte hat die niedrigste Biegefestigkeit und Bruchfestigkeit, Stahl der 300er-Serie hat die höchste Biegefestigkeit und Duktilität (Bruchfestigkeit). Bei den Nadeln sollen laut EASSI (European Association of the Surgical Suture Industry) Standards eingehalten werden, bei Nichteinhaltung der Qualität sollten sie nicht verwendet werden. So werden Verfärbungen, Flecken, Vertiefungen (Defekte) auf der Oberfläche, die mit dem bloßen Auge oder unter der definierten Vergrößerung erkennbar sind, als **Nadelfehler**

Nadelfehler

eingestuft [3]. Partikel auf der Oberfläche, die unter der festgelegten Vergrößerung sichtbar sind, stellen einen Defekt dar [3].

## Aufbau einer chirurgischen Nadel

Eine chirurgische Nadel können wir beschreiben mit den **Abschnitten**

Abschnitten

Nadelspitze, Nadelkörper, Nadeldurchmesser, Nadelradius, Armierzone bzw. Öhrzone, Nadellänge und Sehnenlänge (s. Abb. 1).

Mit der Sehnenlänge wird die Gerade zwischen Nadelspitze und Nadelende/Öhr bezeichnet. Die Nadellänge gibt die Strecke von der Spitze entlang des Verlaufs der Nadel bis zur Armierzone/Öhr an. Der Nadelkörperdurchmesser und der Durchmesser des eingesetzten Fadens sollten möglichst identisch sein, um das Trauma für das Gewebe wie Gewebeeinreißung und Blutung durch größeren Fadendurchmesser zu vermeiden. Am Nadelkörper wird der Nadelhalter angesetzt (s. unten).

## Atraumatische und traumatische Nadeln

Grundsätzlich werden bei den Nadeln 2 Grundformen unterschieden: atraumatische und traumatische **Nadel-Faden-Verbindungen**

Nadel-Faden-Verbindungen.

Die Verbindung zwischen Nadel und Faden erfolgt entweder über ein Öhr oder öhrlos. Die traumatischen Nadeln besitzen zur Fadenaufnahme ein Öhr.

### Traumatische Nadeln

**Figure 2:**
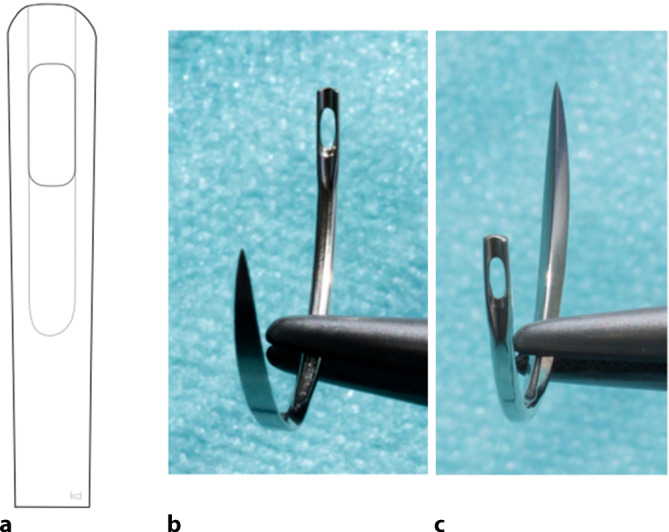

**Figure 3:**
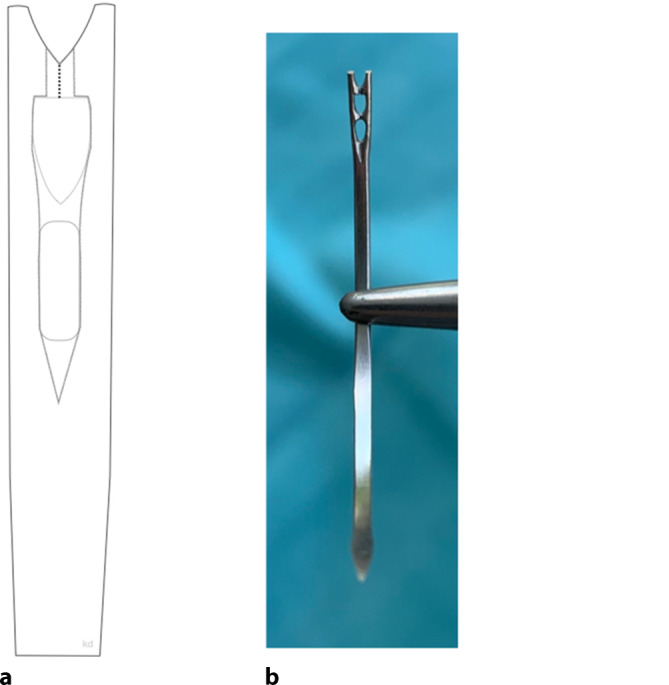

Bei den traumatischen Nadeln wird das Nahtmaterial in das Auge oder Öhr, z. B. Langlochöhr (Abb. 2), eingefädelt oder durch einen Schlitz, ein Federöhr bzw. Schlitzöhr (Abb. 3) hineingezogen. Das **Langlochöhr**

Langlochöhr

erinnert sehr an Haushaltsstopfnadeln. Der Faden wird bei diesem Vorgang nicht geschädigt. Beim **Schlitzöhr**

**Figure 4:**
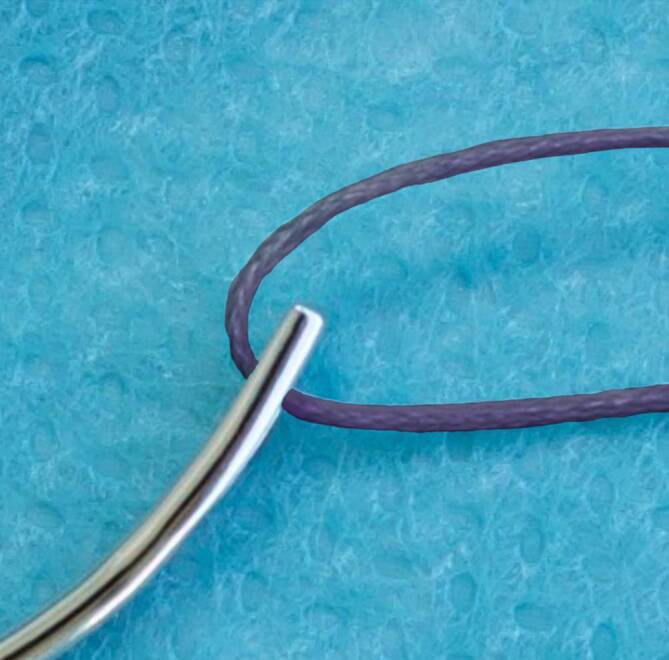

Schlitzöhr

wird das Nahtmaterial durch den Schlitz in das Öhr gezogen. Beim Durchziehen des Fadens durch den geschlitzten Teil des Öhrs können hohe Reibungseffekte entstehen und zur Abrasion des Nahtmaterials im Schlitz führen. Bei beiden Varianten liegt der Faden also immer doppelt auf einer gewissen Strecke hinter dem Öhr (Abb. 4).

Das Nahtmaterial löst deshalb im Gewebe einen größeren **Reibewiderstand**

Reibewiderstand

aus, der teilweise auch als Sägeeffekt bezeichnet wird. Beim Durchgang des doppelsträngigen Nahtmaterials in der Öse der Nadel entsteht beim Eintritt in und beim Ziehen durch das Gewebe ein größeres Gewebetrauma, deshalb wird von traumatischen Nadeln gesprochen. Auch bei geöhrten Nadeln sollten Fadendurchmesser und Nadelöhr aufeinander abgestimmt sein. **Nadeln mit Öhr**

Nadeln mit Öhr

werden teilweise als wirtschaftlich(er) angesehen, da die Nadeln einige Male wiederverwendbar sind. Dies wird aber auf Kosten von abnehmender, also geringerer Schärfe und größerer Traumatisierung des durchstochenen Gewebes in Kauf genommen. Aus hygienischen Gründen sollten diese Nadeln eigentlich nicht mehr regulär zum Einsatz kommen. Auch erfordert das Einfädeln mehr Zeit als das Anreichen einer mit Faden armierten Nadel, sodass der ökonomische Faktor Materialersparnis durch Personalkosten und Operationssaalkosten aufgefressen wird. Zusätzlich sollte das Verletzungsrisiko des Personals beim Einfädeln nicht außer Acht gelassen werden.

### Atraumatische Nadeln

**Figure 5:**
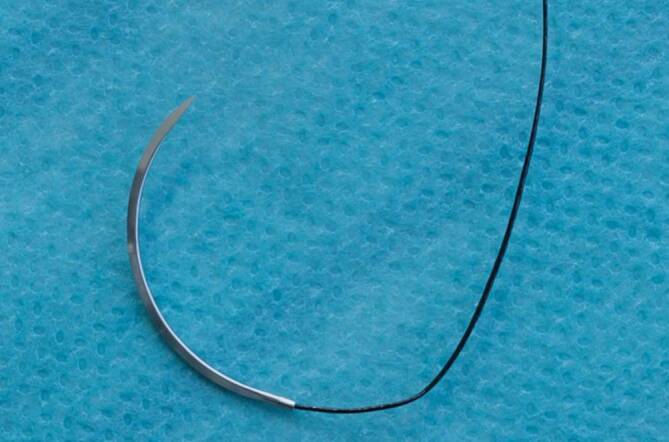

Atraumatische Nadeln, auch **gestauchte Nadeln**

gestauchte Nadeln

genannt, haben kein Öhr [4]. Die Fadenaufnahme wird mittels mechanischer oder Laserbohrung in die Armierungszone eingebracht [2]. Die Laserbohrung ermöglicht längere Bohrkanäle und damit bessere Verankerungen der Fäden [2]. Der Faden ist ohne wesentlichen Durchmesserunterschied (Abb. 5) in der Nadel verquetscht, verklebt, gelasert. Das etwas gestauchte Ende verbindet die Nadel mit dem Nahtmaterial. Nahtmaterial und Nadel können so ohne eine zusätzliche Gewebeverletzung, als der Nadelstich selbst verursacht, ins Gewebe geführt werden. Der Durchmesser von Nadel und Nahtmaterial ist geringer als bei der geösten Nadel. Der Faden ist werkseitig fest mit der Nadel verbunden. Die stufenlosen Verbindungen von Nadel und Faden werden „atraumatisch“ genannt [5]. Eine Sonderform bei den atraumatischen Nadeln sind die **Abreißnähte**

Abreißnähte.

Hier wird durch kurzen Zug an der sicher im Nadelhalter gehaltenen Nadel der Faden aus seiner Verankerung in der Armierungszone mit einer Kraft von 3–10 N herausgezogen [5].

## Nadelkrümmung/-radius

Grundsätzlich werden gerade von gebogenen Nadeln unterschieden. Im Detail werden wir die geraden Nadeln nicht detailliert abhandeln, da sie sich nur in der Nadelform, aber nicht an der Armierungszone unterscheiden. Die Nadelkrümmung oder Nadelbiegung wird ursprünglich bezogen auf den Kreisdurchmesser in Achtelbruchanteilen. Oder anders ausgedrückt ist dies der Radius von der Kreismitte zum Nadelkörper, wenn die Krümmung der Nadel zu einem vollen Kreis fortgesetzt würde.

**Figure 6:**
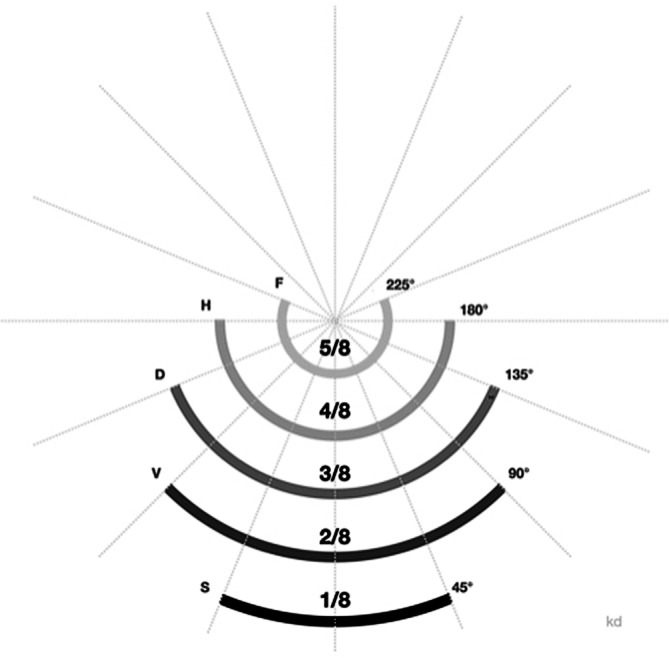

In Deutschland wird die jeweilige Bruchangabe mit einem Buchstaben kodiert, (Abb. 6). Mit **gekrümmten Nadeln**

gekrümmten Nadeln

wird weniger Platz zum Nähen benötigt als mit **geraden Nadeln**

geraden Nadeln;

1/4, 3/8, 1/2 und 5/8 sind die am häufigsten verwendeten Krümmungen. Die Krümmung programmiert den Weg der Nadel durch das Gewebe vor. Die Schlitten- oder Skinadel werden nur der Vollständigkeit halber erwähnt. Diese finden in Orthopädie und Unfallchirurgie eigentlich keine Anwendung, sondern eher im laparoskopischen Bereich.

Nadeln mit einer Krümmung von 135° (3/8) eignen sich gut für Inzisionen, Platzwunden etc., die leicht zugänglich sind. Hier ist nur eine geringe **Handgelenkdrehung**

Handgelenkdrehung

erforderlich, um die Nadel durch das Gewebe zu führen [2]. In der Tiefe des Operationsgebietes, z. B. Becken, ist die 3/8-Nadel ungünstig, da die Handgelenkdrehung in der Regel nicht ausreicht, um die Nadelspitze wieder aus den Gewebeschichten herauszuführen [2]. Die Nadelspitze bleibt im Gewebe und kann in dem tiefen kleinen Operationsfeld nur schwierig durch Nachsetzen des Nadelhalters getastet werden. Bei diesem Tastmanöver mit den leicht geöffneten Branchen des Nadelhalters wird das Gewebe häufig traumatisiert. Bei **tiefem Operationsfeld**

tiefem Operationsfeld

ist eine 1/2 (180°)-Nadel günstiger im Handling, da auch bei anatomisch begrenzter Handgelenkdrehung die gesamte Nadel durch das Gewebe geführt und damit die Nadelspitze gefasst werden kann [2]. In der **Mikrochirurgie**

Mikrochirurgie

kommen Nadeln mit einem 90°-Krümmungswinkel (1/4-Nadeln) häufiger zum Einsatz.n

Es kann festgestellt werden, dass die Krümmung der Nadeln immer mehr gebogen sein sollte, je weniger Platz im Operationsfeld vorhanden ist. Je stärker die Nadel gebogen ist, desto näher liegen Einstich- und Ausstichstelle zusammen. In Orthopädie und Unfallchirurgie sind 3/8-Nadeln häufig in Verwendung für Haut, in der Handchirurgie, zur Naht von Faszien, Muskeln und Subkutangewebe; 1/4-Nadeln werden verwendet in der Mikrochirurgie und zur Fasziennaht. Gerade Nadeln werden bei Sehnennähten verwendet.

### Merke

Während des Nähvorgangs sollte der Operateur beachten, dass er Nadelhalter und Nadel in einem dem Nadelradius entsprechenden Kreis geführt wird.

## Zusätzliche Nadelspezifikationen

Bei der Nadelstärke werden dünner Nadelkörper (f), starker Nadelkörper (s), sehr starker Nadelkörper (ss), Break-off-Nadel (v) und schwarze Nadel (b) unterschieden.

## Nadellänge

Auf dem Markt existiert ein Angebot verschiedenster Nadellängen von zahlreichen Herstellern für die verschiedenen Einsatzgebiete. Die Nadellänge wird gemessen entlang der Nadelkrümmung von der Spitze bis zur Armierungszone (Abb. 1).

## Nadelspitzen

Die Nadelspitze erstreckt sich bis zum maximalen Querschnitt des Nadelkörpers [2, 4]. Die Nadelspitze dient zur Perforation bzw. **Penetration des Gewebes**

Penetration des Gewebes.

**Figure 7:**
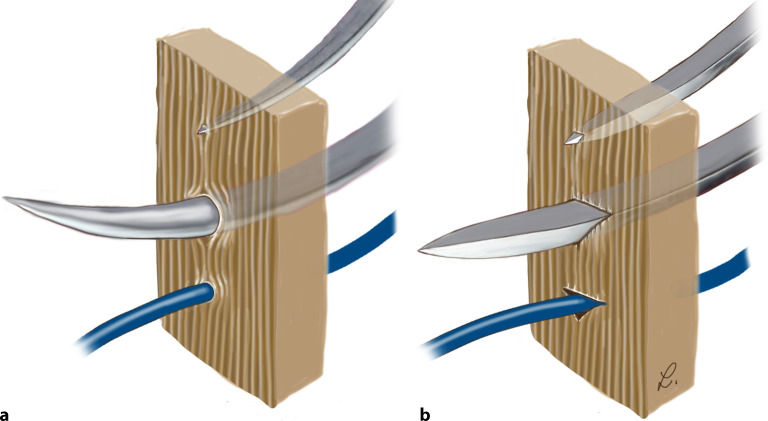

Es werden grundsätzlich stumpfe von spitzen bzw. schneidenden Spitzen unterschieden. Der grundsätzliche Unterschied ist, dass Nadeln mit schneidenden Spitzen das Gewebe durchtrennen, während stumpfe, z. B. konische Nadeln das Gewebe auseinanderdrängen (Abb. 7).

### Stumpfe Nadelspitzen

**Figure 8:**
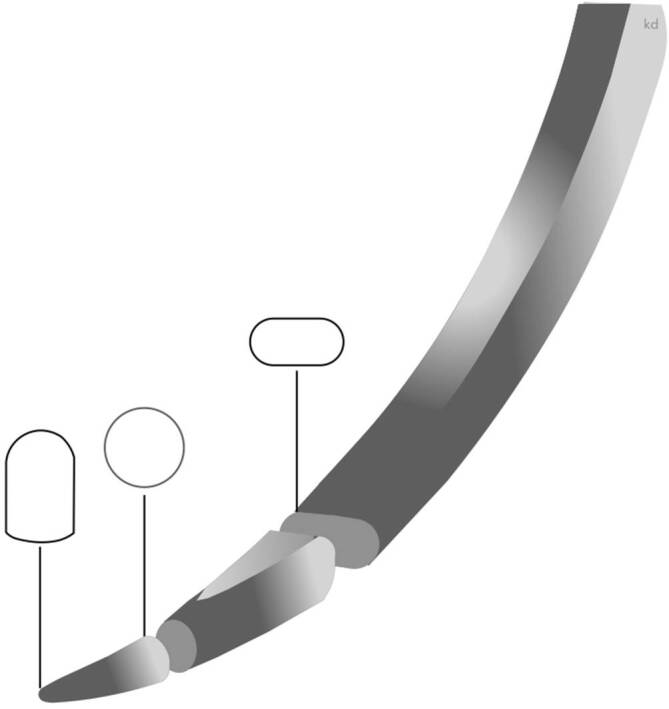

Nadeln mit stumpfer Spitze (Abb. 8) drängen das Gewebe eher auseinander. In weichem, eher brüchigem Gewebe können sie gut eingesetzt werden. Stumpfe Nadeln verletzten auch weniger dicht übereinanderliegende Gewebe, wie z. B. den Darm bei der Peritoneumnaht. Stumpfe Nadeln perforieren schlechter die Operationshandschuhe und reduzieren so das Risiko durch **intraoperative Nadelstichverletzungen**

intraoperative Nadelstichverletzungen.

Der Nadelkörper kann verschiedene Formen von rund bis rechteckig annehmen.

### Konische Nadeln

Konische Nahtnadeln werden aufgrund ihrer Form und der spitzen oder stumpfen Spitze auch als **Rundkörpernadeln**

Rundkörpernadeln

 (R) bezeichnet. Der Nadelkörper ist rund und verjüngt sich ohne Stufe, also konisch, zu einer Spitze. Diese Nadeln haben keine scharfe, schneidende Kante. Diese Nadeln durchdringen das Gewebe durch Verdrängung, Spreizung und Dehnung, ohne zu schneiden [6]. Nach dem Einführen der Nadel schließt sich das Gewebe fest um das Nahtmaterial, das wiederum eine Art dichte Barriere bildet, die verhindert, dass Verunreinigungen von außen in die Wunde gelangen. Von der Spitze geht das Material über in einen runden, ovalen oder eher rechteckigen Nadelkörper. Die **Schärfe**

**Figure 9:**
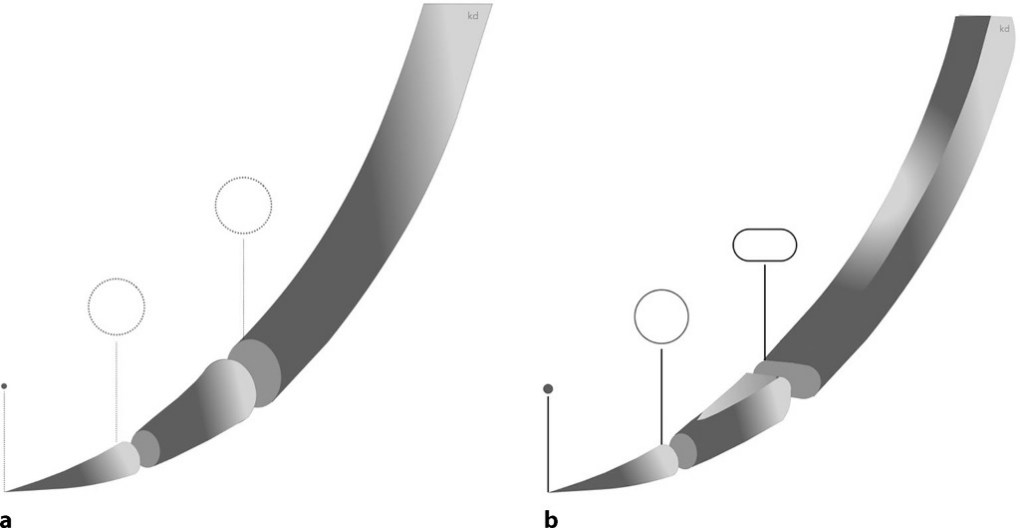

Schärfe

wird durch das Kegelverhältnis (8–12:1) [2] und den Spitzenwinkel (20–35°) bestimmt [6]. Die Nadel ist schärfer, wenn sie ein höheres Kegelverhältnis und einen niedrigeren Spitzenwinkel hat [6], oder einfach ausgedrückt, je länger und schlanker der Kegel der Nadelspitze zuläuft, desto besser penetriert die Nadel das Gewebe. Die Spitzen dieser Nadeln reichen von sehr spitz zulaufend (Abb. 9) bis abgerundet oder stumpf (Abb. 8).

Die **Kegelspitznadeln**

Kegelspitznadeln

(Taper-point-Nadeln) (Abb. 9) werden zum Nähen von Geweben verwendet, die leicht zu durchdringen sind, also von weichem Gewebe wie Gefäß‑, Faszien- und anderem weichen Gewebe [2]. Sie eignen sich nicht für Haut- und Subkutangewebe [4].

#### Merke

Bei Unsicherheit, ob eine spitze oder eine schneidende Nadel gewählt werden soll, sollte die Wahl auf eine Kegelspitznadel für alles außer für Hautnähte fallen. Verschiedene Nadeldurchmesser werden angeboten.

### Scharf schneidende Nadeln

Eine **Schneidnadel**

Schneidnadel

hat mindestens 2 gegenüberliegende Schneidkanten [2]. Heute sind die Spitzen häufig dreieckförmig [6]. Wenn **Schneidkantennadeln**

Schneidkantennadeln

3 Schneidkanten haben, kategorisiert die Position der dritten Schneidkante die Nadel entweder als konventionelle oder als umgekehrte (reverse) Schneidkantennadel [2]. Scharfe Nadeln durchstechen und durchschneiden entsprechend ihrem Durchmesser das Gewebe. Der nachfolgende Nadelkörper spreizt oder schneidet das Gewebe. Die Spitze schneidet den Weg durch das Gewebe. Es werden verschiedene scharfe Spitzenkonfigurationen unterschieden.

## Kegelschneidnadeln

Werden konische Nadeln mit einer scharfen Spitze, 3 bis 4 Schneiden, versehen, so erhalten die Nadeln folgende Eigenschaften: Beim Eintritt schneiden sie, und dann verdrängen sie das Gewebe, sog. **Taper-cut-Nadeln**

**Figure 10:**
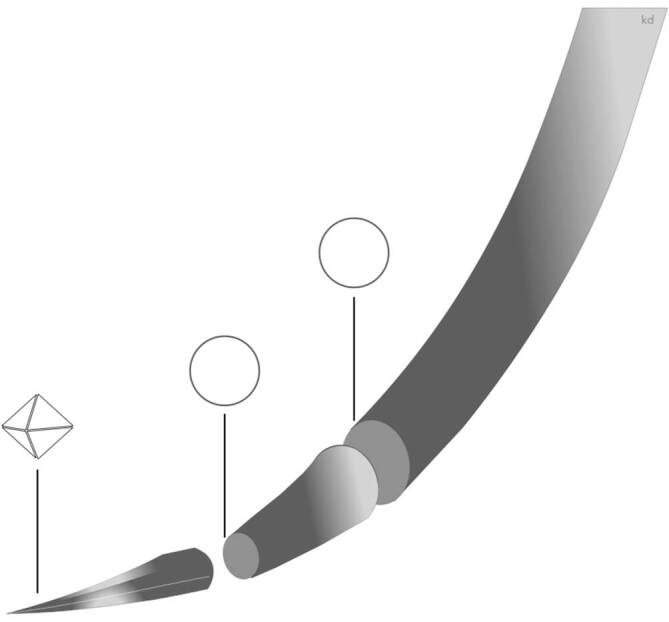

Taper-cut-Nadeln

(Abb. 10). Sie werden in zähem, schwer zu durchdringendem Gewebe verwendet. Sie finden Einsatz zur Hautnaht und für subkutanes Gewebe. In der Gefäßchirurgie eignen sie sich für sklerotische Gefäße, da hier das Gefäß nicht einreißt [2].

### Schneidnadeln

#### Konventionell schneidende Nadeln

**Figure 11:**
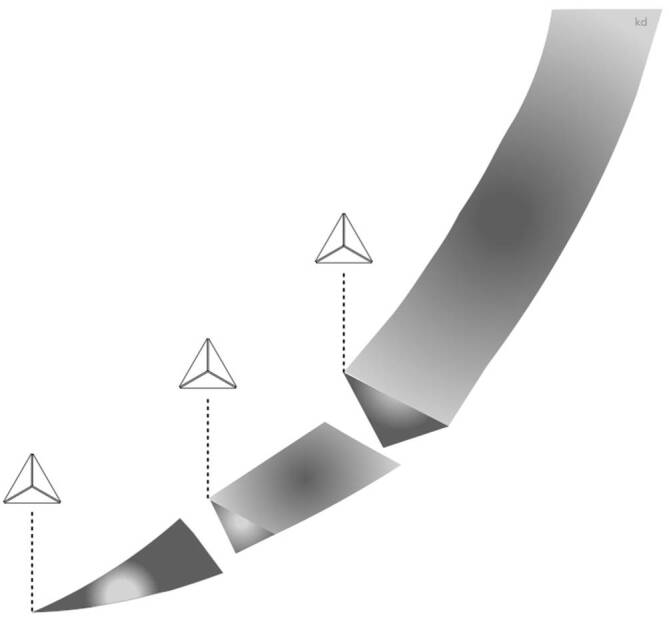

Konventionell schneidende Nadeln bestehen aus einer Spitze, einem dreieckig geformten Körper und 3 geschliffenen Kanten, wobei 2 der Schneiden auf gegenüberliegenden Seiten liegen und die dritte Kante auf der Innenseite, der Konkavität, der Kurve liegt [4]. Sowohl bei geraden als auch bei gebogenen Nadeln geht die Form von einer dreieckigen Schneide meist in einen etwas abgeflachten Restkörper über (Abb. 11).

Konventionell schneidende Nadeln haben im Bereich des Nadelkörpers meist einen **dreieckigen Querschnitt**

dreieckigen Querschnitt.

Durch zusätzliche Innenwölbung der Dreikantschneide bei den Nadeln soll eine Verstärkung der Schneidkraft erreicht werden, diese Nadeln werden als **Präzisionsschnitt-Nadeln**

Präzisionsschnitt-Nadel

vermarktet.

Die Verwendung einer Nadel mit konventioneller Schneidkante hinterlässt ein Loch, das Gewebe ist zerschnitten oder zerrissen [2]. Da sich die dritte Schneide an der inneren, konkaven Krümmung der Nadel befindet, verursacht die innere Schneide einen linearen Schnitt, der senkrecht und nahe an der Inzision liegt, gegen den die Naht eine Wundverschlusskraft ausübt, die letztendlich das Gewebe durchschneiden kann [2].

#### Umgekehrt schneidende Nadeln

**Figure 12:**
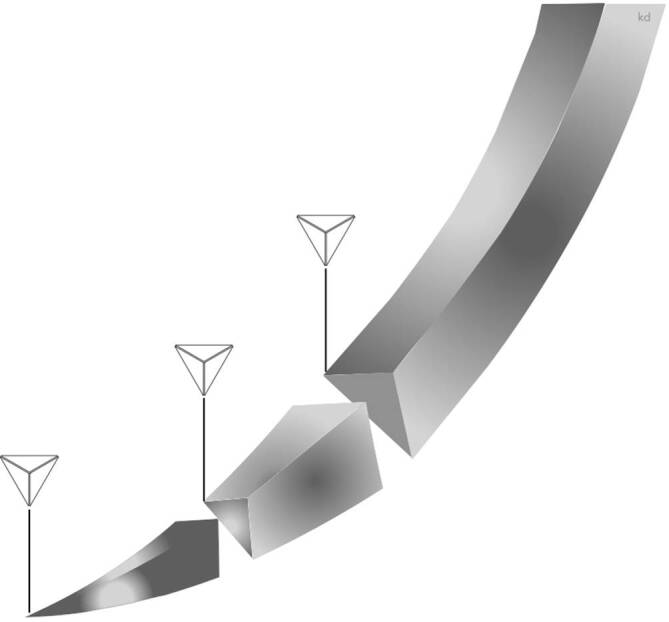

Bei dieser Nadelkonfiguration (Abb. 12) befindet sich die schneidende scharfe Kante an der konvexen Außenseite der Krümmung. Diese Nadeln sind stärker als die normalen Schneidnadeln. Von ihnen geht ein geringeres Risiko aus, das Gewebe zu zerschneiden [6]. **Außenschneidende Nadeln**

Außenschneidende Nadeln

haben den Vorteil, dass beim Rechtshänder das Trauma im Stichkanal minimal gehalten werden kann. Der Hauptdruck beim Durchstechen liegt am Innenbogen [5]. Die Schneide bleibt so von der Wunde und der Zugrichtung entfernt, was die Neigung der Nadel und des Nahtmaterials, durch das Gewebe zu reißen, verringert und gleichzeitig eine einfache Nahtpassage ermöglicht. Beim Knoten des Fadens kann ein Einschneiden des Fadens ins Gewebe besser verhindert werden als bei innengeschliffenen Nadeln [5]. Dies lässt sich damit erklären, dass, wenn die Umkehrschneidnadel die Haut durchschneidet, sie keine Inzision erzeugt, sondern eine breitere Gewebewand, gegen die das Nahtmaterial seine Wundverschlusskraft ausübt [2]. Diese Gewebewand widersteht dem Durchtrennen der Naht.

Umgekehrt schneidende Nadeln sind speziell für zähes, schwer zu durchdringendes Gewebe wie Haut, Sehnenscheide oder Mundschleimhaut. Sie werden u. a. in der **plastischen Chirurgie**

plastischen Chirurgie

eingesetzt, da durch geringes iatrogenes Trauma geringe Narbenbildung beobachtet wird.

**Seitenschneidende Nadeln**

Seitenschneidende Nadeln,

Spatelnadeln, werden in Orthopädie und Unfallchirurgie selten eingesetzt. Sie wurden mehr für **ophthalmologische Eingriffe**

ophthalmologische Eingriffe

entwickelt. Die dreieckige Geometrie wird durch Abflachung der äußeren konvexen Fläche in eine trapezförmige oder spatelförmige Konfiguration umgewandelt [2]. Durch die Abflachung entsteht auch eine seitliche Verbreiterung (Kobrakopf) des Nadelkörpers [2]. Sie eignen sich aber auch gut zum Nähen der Nagelmatrix [2].

## Nadelkörper

Der längste Teil der Nadel ist der Nadelkörper. Hier fasst der Nadelhalter die Nadel. Und der Nadelkörper erzwingt aufgrund seiner Form und Krümmung die Gewebepenetration entsprechend der Form der Nadel.

**Abgeflachte Nadelkörper**

Abgeflachte Nadelkörper,

meist rechteckige Konfigurationen, erhöhen die Stabilität der Nadel durch den Griff in den Nadelhalterbacken. Die Reibehaftung wird bei einigen Nadeln durch Längsrillen oder Riffelung erhöht. Bei den Nadelkörpern werden Rundkörper (R), rechteckige, von schneidenden (S) und lanzettförmigen (L) unterschieden.

Bei **Rundkörpernadeln**

Rundkörpernadeln

 (R) geht die konische Spitze in den runden Nadelkörper über. Teilweise sind die Rundkörpernadeln abgeflacht, um einen besseren Halt der Nadel im Nadelhalter zu gewährleisten. Diese Nadeln eigenen sich z. B. für Muskulatur, subkutanes Fettgewebe, Pleura u. a.

## Nadellänge

Die Nadellänge wird entlang der Nadelkrümmung gemessen von der Spitze bis zur Armierungszone (Abb. 1). Gängige Nadellängen sind 6 mm, 8 mm, 12 mm, 18 mm, 22 mm, 30 mm, 35 mm, 40 mm, 50 mm.

## Nadelstärke/-durchmesser

Der Nadeldurchmesser wird nach EASSI (The European Association of the Surgical Suture Industry) Standard Classification [7] angegeben. So wird für die Klassifikation 1,5 ein Durchmesser von 0,15–0,199 mm angegeben. Kleinere Durchmesser darunter werden als Mikronadeln eingestuft.

## Penetrationskraft

Bei geraden Nadeln wird eine lineare Kraft zur Penetration des Gewebes ausgeübt. Bei gebogenen oder gekrümmten Nadeln führt die Rotationskraft zum Penetrieren oder Verdrängen des Gewebes [8]. Durch **Beschichtungen**

Beschichtungen

der Nadel, sog. Coating, z. B. mit Silikon, Glyconat u. a., können die Gleitfähigkeit [1] und damit das Penetrationsverhalten der Nadel verbessert werden [9].

## Nomenklatur

Eine einheitliche Nomenklatur der Nadeln wird von verschiedenen Herstellern nicht genutzt. Identische Nadeln werden von den Herstellern mit unterschiedlichen Codes angeboten. Die EASSI propagiert zwar einen Standard für eine gemeinsame Klassifizierung für die Identifizierung und Auswahl einer chirurgischen Nadel ([10]; Tab. 1). Die Nomenklatur der Nadeln variiert bei den unterschiedlichen Nadelherstellern teilweise sehr stark ([4]; s. zusätzliche Tabelle online).

**Table 1:** 

	Bedeutung	Englisch
*Kode 1*		
F	5/8	–
H	1/2	–
D	3/8	–
V	1/4	–
K	Halb gekrümmt	Half curved
A	Asymptotisch	Asymptotic
G	Gerade	Straight
*Kode 2*		
Q	Quadratischer Nadelkörper	Square bodied
R	Rundkörpernadel	Round-bodied
S	Schneidender Nadelkörper	Reverse cutting
L	Lanzetten (Spatula)-Nadel	Spatula needle
*Kode 3*		
L	Lanzettspitze	Lancet point
M	Mikrospitze/-kurzschliff	Micro point
MP	Präzisionsspitze	–
m	Mikronadel	–
mV	Mikrogefäßnadel	–
S	Schlanker Anschliff	Slim point
T	Trokarspitze	Trocar point
K	Kurze innenliegende Schneide	Taper cutting
SP	Spatelnadel	Spatula needle
N	Stumpf	Blunt
C	Kurze schneidende Spitze	–
f	Nadelkörper dünn	–
s	Nadelkörper, starke Ausführung	–
v	Break-off-Nadel	–
b	Schwarze Nadel	–

*EASSI* European Association of the Surgical Suture Industry, *Kode 1* Nadelkrümmung, *Kode 2* Querschnitt, *Kode 3* Nadelspitze, *Kode 4* Nadellänge

Um eine übersichtliche Nadelnomenklatur zu ermöglichen, die dem Operateur in anschaulicher Weise Auskunft über Form, Ausführung und Länge der Nadel gibt, existiert eigentlich ein sog. **Nadelcode**

Nadelcode,

zumindest im deutschsprachigen Bereich: Der 1. Buchstabe bezieht sich auf die Form der Nadelkrümmung, der 2. auf den Querschnitt, und der 3. gibt Auskunft über die Ausführung der Nadelspitze. Die Zahl hinter dieser Buchstabenkombination gibt die Länge der Nadel in gestrecktem Zustand wieder [5]. Ein weiterer Buchstabe gibt besondere Eigenschaften wieder (s stark, ss extrastark, d schlank, f flach).

Eine 3/8-Nadel mit Rundkörper und scharfer Spitze mit einer Länge von 24 mm hätte den Code: DRT24.

Vergleicht man die Nomenklatur für chirurgische Nadeln im deutschsprachigen Raum, fällt auf, dass amerikanische Hersteller sich in der Kodierung und Nomenklatur der Nadeltypen von den europäischen deutlich unterscheiden. Aber auch innerhalb der amerikanischen Hersteller variiert die Nomenklatur, sodass auch hier Vergleiche zumindest bei der Feinspezifikation erschwert sind.

Im angloamerikanischen Raum wurden teilweise funktionsabhängige Bezeichnungen der Nadeln eingeführt (Tab. 2 und zusätzliche Tabelle online).

**Table 2:** 

Abkürzung	Bedeutung	Übersetzung	Einsatz
FS	For skin	Für Haut	Schneidende Nadeln
FSL	For skin large	Für Haut groß	Hautnaht mit größerer Spannung
FSLX	For skin extra large	Für Haut extragroß	Größere Hautnaht
P	Plastic	Plastisch	Für plastisch rekonstruktive Naht Hand und Gesicht – kleine Inzisionen
SH	Small half circle	Kleiner Halbkreis	–
CT	Circle taper	Konische Halbkreis-Nadel	–

## Verpackungsvorgaben, Kennzeichnung

Die Kennzeichnung von Nahtmaterial ist in der EU und weltweit inzwischen geregelt: DIN EN ISO 15223‑1 [11]. Die ISO-15223‑1 bedient sich der Symbole der ISO-7000.

## Sicherheitsmaßnahmen

Folgende Vorsichtsmaßnahmen muss auch der Operateur kennen:

- beim Öffnen der Streifenverpackungen auf Unversehrtheit der Verpackung achten, ansonsten die Nadeln/Nahtmaterial verwerfen,
- Haltbarkeitsdatum überprüfen. Keine Nadeln/Nahtmaterial nach dem Verfallsdatum verwenden,
- ausgepackte Nadeln auf Unversehrtheit prüfen,
- Nadeln mit Defekten aussortieren,
- bei geösten Nadeln die Intaktheit des Öhrs prüfen lassen, damit es nicht zur Fadenabrasion und damit Schädigung des Fadens kommt,
- Nadelstichverletzungen vermeiden. Erst nach Rücknahme von Nadelhalter mit Nadel die neue Nadel-Nadelhalter-Kombination anreichen lassen,
- alle Nadeln in Sammelbox für scharfe Gegenstände sammeln lassen,
- Zählkontrolle immer durchführen (lassen).

## Zusammenspiel Nadelhalter und Nadel

Ein Nadelhalter besteht aus 2 Hebeln, die sich um einen gemeinsamen **Drehpunkt**

Drehpunkt

drehen [12]. Distal des Drehpunkts sind die Backen, häufig Hartmetallbacken [12]. Die Griffe der Nadelhalter sind unterschiedlich konfiguriert.

Die allgemein in Orthopädie und Unfallchirurgie verwendeten Nadelhalter, z. B. Typ Hegar, haben eine **Arretierung**

Arretierung,

ein Schloss, das über eine Rastung am Griff, die konstante Einklemmung der Nadel im Nadelhalter gewährleistet, ohne dass weiterer Druck auf die Branchen des Halters ausgeübt werden muss. Die Branchen sind mit Ringen versehen, in die Daumen und vierter Finger greifen. Die Kraft, die in den Backen entsteht, ist proportional der Länge der Griffe und umgekehrt proportional der Länge der Backen [12].

Die Nadel wird im Nadelhalter festgehalten, indem er zusammengedrückt wird, bis die erste Raste am Schloss einrastet. Der Nadelhalter sollte nicht zu fest angezogen werden, da es sonst zu Beschädigungen sowohl an der Nadel als auch am Nadelhalter kommen kann. Häufig reicht eine Rastung am Schloss des Nadelhalters aus. Das **Klemmmoment**

Klemmmoment

des Nadelhalters ist das Maß für die Kraft, die von den Backen des Nadelhalters auf eine chirurgische Nadel ausgeübt wird [2].

**Figure 13:**
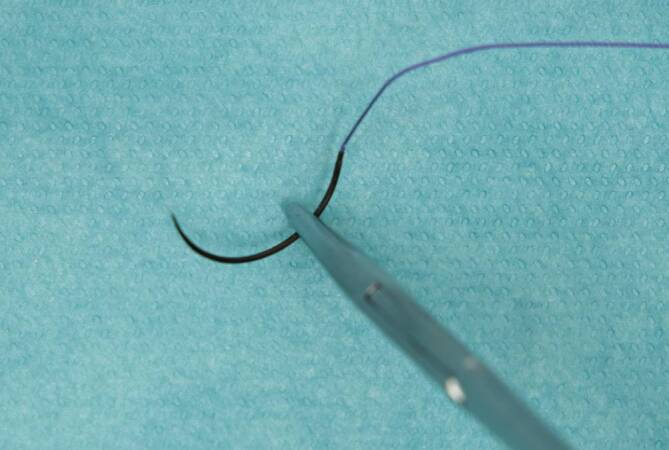

Die Nadel wird im Normalfall senkrecht und in Längsrichtung senkrecht zum Nadelhalter gehalten, dabei sollte die Nadel nicht mit der Spitze des Halters, sondern wenige Millimeter dahinter gefasst werden (Abb. 13).

Die **gerieften Backen**

gerieften Backen

im Maul des Nadelhalters halten die Nadeln. Die Haltebacken aus Hartmetall sind resistenter gegen Verschleiß und Abnutzung. Diese Nadelhalter haben als Zeichen der Hartmetalleinlage normalerweise einen goldfarbenen Griff.

**Figure 14:**
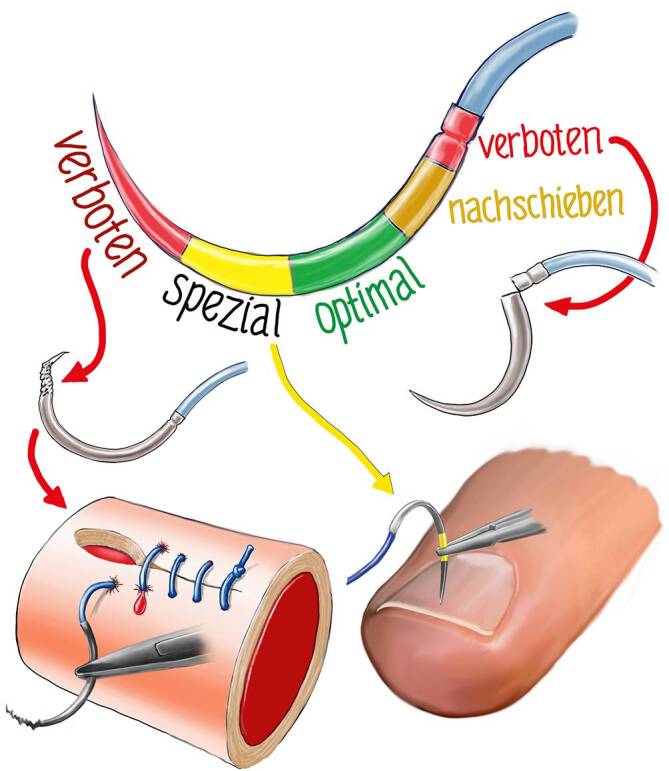

Die Nadel wird optimal, von der Nadelspitze an gerechnet, etwa im mittleren Drittel (Zone grün) gefasst. Zonen, die nicht gefasst werden sollten, sind die Nadelspitze und die Armierungszone (rote Zone) (Abb. 14). Die Nadelspitze kann durch das Fassen mit dem Nadelhalter beschädigt werden, verbiegen oder brechen [2]. Mit der dann geschädigten oder gekröpften Spitze kann nicht mehr atraumatisch genäht werden. Dies zeigt sich besonders bei Gefäßnähten (s. Abb. 14).

### Merke

Der Nadelhalter sollte auf keinen Fall in der Armierungszone die Nadel fassen, da es beim Schließen der Backen zu Verbiegungen des Materials oder zum Bruch kommen kann.

In **Spezialfällen**

Spezialfällen,

wie z. B. der Perforation eines Fingernagels, kann die Nadel direkt hinter der Spitze (gelbe Zone) gefasst werden, um eine bessere Perforation dieses harten Materials zu ermöglichen. Bei derbem oder hartem Material reicht die normale Haltung des Nadelhalters nicht aus. Der Nadelhalter muss einerseits die Nadeln fest fassen, aber auch die Handhaltung muss entsprechend geändert werden (s. unten, Abb. 17 und 19).

**Figure 15:**
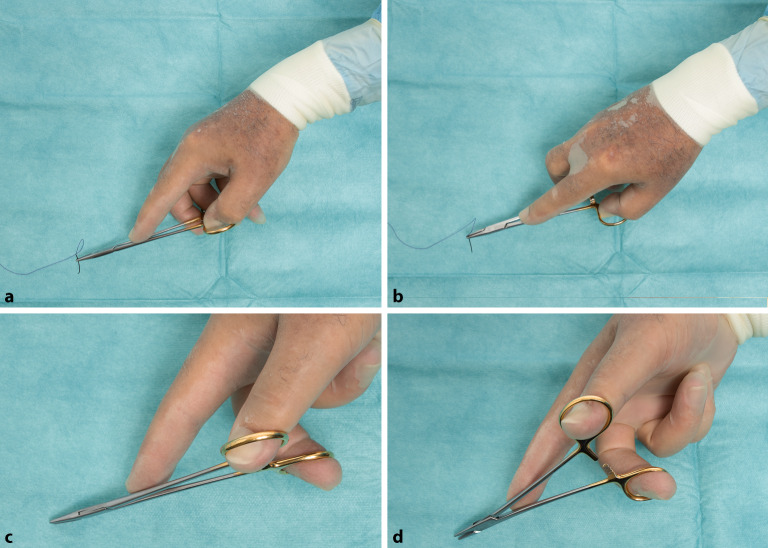

In manchen Situationen muss die Nadel nachgeschoben werden. Hierzu wird die Nadel mit dem Nadelhalter in kleinen Schritten geschoben, bis man die optimale Zone erreicht.

Sind die Nadeln abgeflacht, werden sie am sichersten im abgeflachten Teil gefasst. Abgenutzte Backen lassen einen festen korrekten Grip häufig vermissen, sodass sich die Nadel während des Durchführens im Gewebe im Maul des Nadelhalters verdrehen kann. Die Haltekraft ist eher abhängig von der Riffelung der Backen als von der Oberflächenbeschaffenheit der Nadeln [2, 12].

Die **Größe und Länge**

Größe und Länge

des Nadelhalters sollten hinsichtlich Operationsschrittanforderung und Nadelgröße korrekt gewählt werden. Die Backen des Nadelhalters müssen auf die Größe der Nadel abgestimmt sein, um diese sicher zu halten und ein Wackeln, Drehen und Kippen zu verhindern. Es muss ein Gleichgewicht zwischen Nadelhalterlänge und Nadelgröße bestehen.

### Merke

Je größer die Nadel, desto größer sollte der Nadelhalter gewählt werden.

**Zu klein dimensionierte Nadelhalter**

Zu klein dimensionierte Nadelhalter

lassen die Nadel leicht um die Längsachse rotieren [6]. Ein abgeflachter oder mehr ovaler Nadelquerschnitt verbessert oft sowohl den Oberflächenkontakt mit den Nadelhalterbacken [12] als auch das Biegemoment der Nadel [6].

Werden **zu große Nadelhalter**

zu große Nadelhalter

bei kleinen Nadeln gewählt, kann die Nadel beschädigt werden. Aber es kann auch durch zu große Nadelhalter zum Abflachen des Nadelradius kommen. Mechanisch kommt es beim Verklemmen der Nadel in den Backen des Nadelhalters zur Reibehaftung zwischen den beiden Partnern. Mechanisch gesehen, muss das Klemmmoment des Nadelhalters kleiner sein als das Elastizitätsmodul der Nadel, sonst verbiegt sich die Nadel und kann schließlich brechen [6]. Wiederholte Beschädigungen durch den Nadelhalter können einerseits zur Beschädigung der Nadeloberfläche und damit zu erhöhter Reibung im Gewebe führen, andererseits kann dies ebenfalls zum Bruch der Nadel führen. Eine **falsche Platzierung**

falsche Platzierung

der Nadel im Nadelhalter kann also zu einer verbogenen Nadel, einer schwierigen Penetration in die Haut oder einem unerwünschten Eintrittswinkel in das Gewebe führen.

Außerdem nimmt eine verbogene Nadel einen relativ traumatischen Weg durch das Weichteilgewebe und kann zu vermehrten Weichteilverletzungen führen. Zu scharfen Kanten der Maulfläche können zum Bruch insbesondere von monofilem Nahtmaterial beim Knoten führen.

## Wie halte ich den Nadelhalter?

### Daumen-Ringfinger-Griff

Der Nadelhalter wird gehalten, indem der Daumen und der vierte Finger in die Ringe der beiden Branchen gelegt werden und der Zeigefinger auf den Drehpunkt des Nadelhalters gelegt wird, um für Stabilität zu sorgen (Abb. 15/Video 1 online). In der Ausgangsstellung dringt das Daumenendglied nur wenig in das Nadelhalterohr ein, der Zeigefinger liegt zur Führung des Nadelhalters ca. am Übergang zum vorderen Drittel. Auch der **Mittelfinger**

Mittelfinger

wird mit etwa zwei Dritteln der Fingerkuppe in den entsprechenden Ring des Halters gelegt.

Der **Zeigefinger**

Zeigefinger

spielt bei der Führung des Nadelhalters eine entscheidende Rolle. Mit korrekter **Drei-Punkte-Stabilisierung**

**Figure 16:**
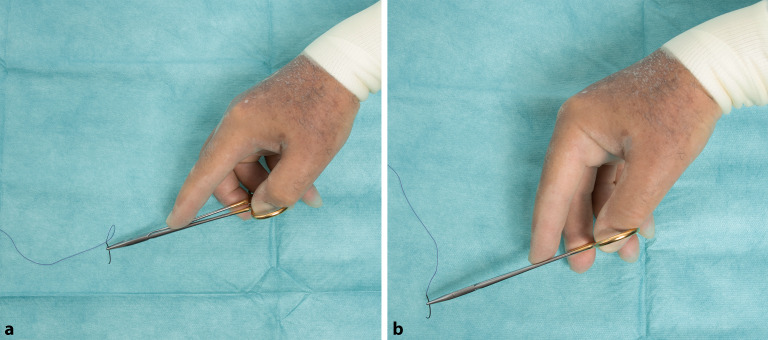

Drei-Punkte-Stabilisierung

durch den Zeigefinger ist neben dem sicheren Halt auch die ungehinderte Drehung des Nadelhalters erreicht (Abb. 16).

### Daumenballengriff

**Figure 17:**
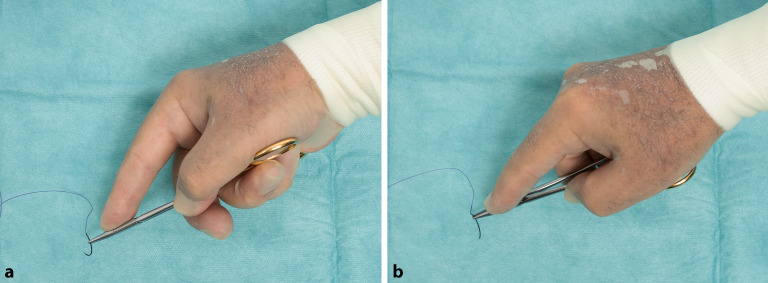

**Figure 18:**
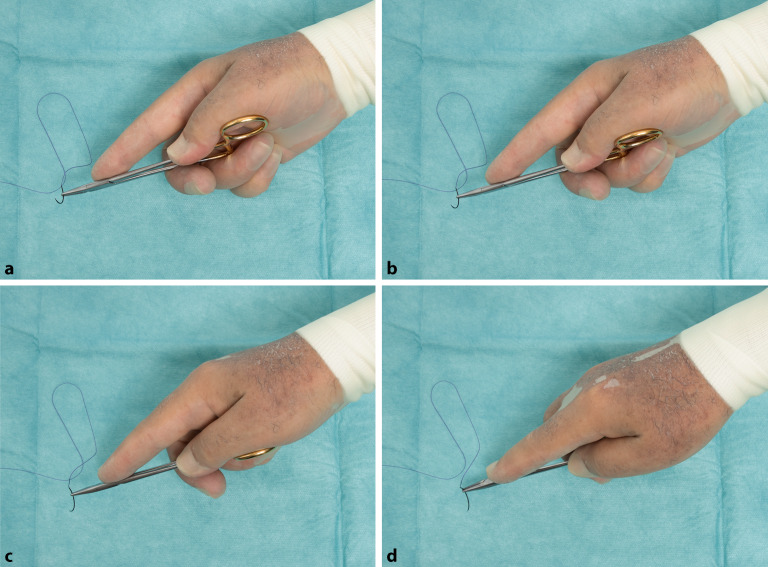

Alternativ kann der Nadelhalter auch in der Handfläche gehalten werden, um die Kraft und Fingerfertigkeit, z. B. bei Periost- oder Sehnennähten, zu erhöhen. (Abb. 17/Video 2 online). Beim Daumenballengriff liegt das Ende des Nadelhalters frei in der Hand und wird mit Mittelfinger und Zeigefinger gegen den Daumenballen gedrückt (Abb. 18).

### Handflächengriff

**Figure 19:**
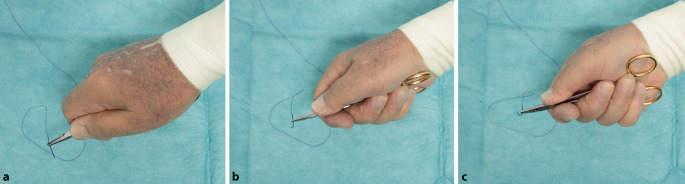

Der Nadelhalter wird in der Handfläche gehalten, was eine **größere Kraftübertragung**

größere Kraftübertragung

bei zähem Gewebe ermöglicht (Abb. 19). Bei dieser festen Fixierung des Nadelhalters in der Hand erfolgt die Drehung mit der ganzen Hand.

## Nadelhalterführung durch Linkshänder

**Figure 20:**
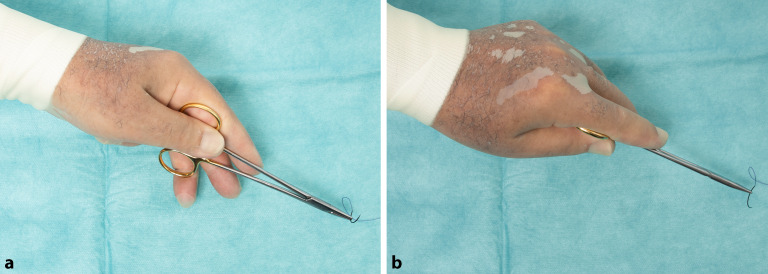

In den meisten Kliniken werden für linkshändische Kollegen und Kolleginnen keine separaten Instrumente vorgehalten. Da die meisten Nadelhalter (wie auch Scheren) für Rechtshänder konstruiert sind, gilt es für Linkshänder, gewisse Punkte zu beachten. Der wesentlichste Punkt ist, dass in der Standardanwendung keine Fingerbeere in die Nadelhalterringe geführt werden sollte. Dies führt aufgrund des Nadelhalterverschlussmechanismus dazu, dass der Nadelhalter leicht aufspringen kann. Deshalb wird, ähnlich wie beim Handflächengriff, der Nadelhalter lediglich durch den Daumen in die Hand gepresst (s. Abb. 20).

## Fazit für die Praxis

- Die ideale chirurgische Nadel sollte starr genug sein, um einer Verformung zu widerstehen, jedoch flexibel genug, um sich zu biegen, bevor sie bricht, so schlank wie möglich sein, um ein Trauma zu minimieren, scharf genug, um das Gewebe mit minimalem Widerstand zu durchdringen, und stabil in einem Nadelhalter sein, um eine genaue Platzierung zu ermöglichen.
- Drei Haupttypen von chirurgischen Nadeln werden unterschieden: Schneidende Nadeln (konventionell, umgekehrt) haben einen dreieckigen Querschnitt und werden für Haut und feste Gewebe verwendet. Konische Nadeln mit spitzer Spitze durchdringen die Gewebe, schneiden aber nicht und werden für weiches Gewebe verwendet. Nadeln mit stumpfer Spitze sind nicht scharf, um Beschädigungen durch die Nadel zu vermeiden und Stichverletzungen zu minimieren.
- Konische Nadeln werden eher bei inneren Gewebeschichten, scharfe Nadeln eher zum Hautverschluss und für derbe Gewebe wie Sehnen und Periost verwendet.

### Supplementary Information








## CME-Fragebogen

Auf was sollte der Operateur während des Nähens achten?

Nadelhalter und Nadel sollen in einem dem Nadelradius entsprechenden Kreis geführt werden

Der Nadelhalter sollte in einem dem Nadelradius entsprechenden Kreis geführt werden

Die Nadelbiegung/-krümmung kann durch die Breite der Nadelhalterbranchen nicht verändert werden

Bei Unsicherheit, ob eine spitze oder eine schneidende Nadel gewählt werden soll, sollte die Wahl immer auf eine schneidende Nadel fallen

je kleiner die Nadel, desto größer wird der Nadelhalter gewählt

In welchem Bereich sollten die Backen des Nadelhaltes regulär die Nadel fassen?

In der Armierungszone

An der Nadelspitze

Im mittleren Drittel

Am Übergang vom mittleren zum hinteren Drittel

Direkt am Fadenansatz

Wie sollte der Nadelhalter beim Daumen-Ringfingergriff gehalten werden?

Der Mittelfinger wird bis zum Mittelgelenk in den Ring der Nadelhalterbranche eingeführt

Der Mittelfinger dringt nur wenig in den Nadelhalterring ein

Der schienende Zeigefinger wird möglichst nah an der Nadel geführt

Der Zeigefinger liegt möglichst an den Nadelhalterringen

Der Daumen wird bis zum Grundgelenk in den Ring eingeführt

Welche Eigenschaften hat eine konische Nadel?

Sie durchschneidet das Gewebe

Sie verdrängt das Gewebe

Sie hat zwischen Spitze und Nadelkörper eine Stufe

Die „Schärfe“ ist abhängig vom Nadelkörper

Sie ist meist gerade

Atraumatische Nadeln…

…haben immer eine geöhrte Armierungszone

…haben einen Kalibersprung zwischen Nadel und Faden

…führen zu einem ausgeprägteren Gewebetrauma als Öhrnadeln

…besitzen keinen Kalibersprung zwischen Nadel und Faden

…sind stabiler als traumatische Nadeln

Was trifft für umgekehrt schneidende Nadeln zu?

Die schneidende scharfe Kante ist an der konvexen Außenseite der Krümmung

Umgekehrt schneidende Nadeln verursachen ein größeres Gewebetrauma

Umgekehrt schneidende Nadeln sollten für leicht zu durchdringendes Gewebe bevorzugt werden

Umgekehrt schneidende Nadeln führen immer zu einer ausgeprägteren Narbenbildung

Sie gibt es nur in großen Radiusdimensionen

Bei der in Deutschland üblichen Nadelkodierung geben die Buchstaben und Zahlen an:

1 Querschnitt 2 Nadellänge 3 Nadelkrümmung 4 Nadelspitze

1 Nadelkrümmung 2 Querschnitt 3 Nadelspitze 4 Nadellänge

1 Nadelspitze 2 Querschnitt 3 Nadellänge 4 Nadelkrümmung

1 Nadellänge 2 Nadelspitze 3 Nadelkrümmung 4 Querschnitt

1 Nadelkrümmung 2 Nadelspitze 3 Nadellänge 4 Querschnitt

Welche Sicherheitsmaßnahmen sind bei Nadeln/Nahtmaterial zu beachten?

Nadeln dürfen, wenn sie trocken gelagert werden, auch nach dem Haltbarkeitsdatum verwendet werden

Nadeln unterliegen bei der Produktion so hohen Anforderungen, dass defekte Nadeln nicht in den Handel kommen

Bei Nadeln mit verbogenen Spitzen unter sterilen Kautelen mit einer Zange die Spitze wieder gerade biegen

Nur bei Unversehrtheit der Verpackung Nadeln/Nahtmaterial verwenden

Nadeln mit Öhr nur einmal verwenden

Durch was kann die Nadelkrümmung unerwünscht verändert werden?

Durch narbiges Gewebe

Durch die Breite der Nadelhalter-Branchen

Durch die Wahl einer zu dünnen Nadel

Die Nadeln sind so stark, dass die Krümmung nicht verändert werden kann

Traumatische Nadeln behalten immer ihre Form

Wo besteht die Gefahr eines Nadelbruches einer traumatischen Nadel mit Feder Öhr?

An der Nadelspitze

An der Stelle wo der Nadelhalter die Nadel hält

Am Übergang Nadel zum Nadelöhr

Sie kann überall brechen

Traumatische Nadeln brechen in der Regel nicht
