# Supplementary material for: Surgical needles in orthopedics and trauma surgery
Source: Oper Orthop Traumatol. 2021 Sep 16;33(5):405–21. [Article in German] doi: 10.1007/s00064-021-00734-7 (PMC8460540; doi:10.1007/s00064-021-00734-7)
Supplement: Supplementary file 1 [file 64_2021_734_MOESM1_ESM.pdf]

Äquivalenztabelle chirurgischer Nadeln in Orthopädie und Unfallchirurgie

Die Nadeln wurden nach Internetrecherche aus den uns zugängigen Unterlagen der Hersteller zusammengestellt. Für Vollständigkeit und Exaktheit kann keine Garantie übernommen werden. Für Korrekturhinweise sind wir dankbar.

Erklärung der Symbole:

Kegelspitze	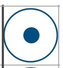
Kegelspitze, abgeflacht	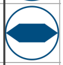
Spatelnadel	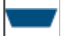
Schneidend, revers	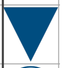
konisch, stumpf	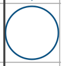
Rundkörper schneidende Spitze, revers	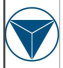
Rundkörper schneidende Spitze, konventionell	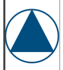
Schneidend Spezial spezial	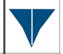
Schneidend Spezial spezialrevers	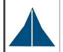
Rundkörper, kurze schneidende Spitze	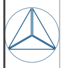
scharf, revers, Mikrospitze	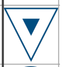
stumpf Rundnadel	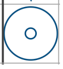

Verwendete Literatur/Internetrecherche:

1. B.Braun B.Braun Nahtmaterial Gut zu wissen. B24601 1218/2/5: www.aesculap.de

2. Dunn DL, Phillips J (2007) Wound closure manual. Ethicon, Somerville, New Jersey

3. Ethicon (2020) Nadel- und Nahtmaterialübersicht 152682-201026-DACH_J11B247. https://www.ethicon.com/emea/epc/search/platform/wound%20closure?lang=de-de

4. Ethicon (2021) Schon Gewusst. J11B061V19: B. BRAUN NAHTMATERIALhttps:// www.bbraun.de › bbraun › AEM2015 › de-de

5. SERAG-Wiessner. Nahtmaterial-Fibel Materialkunde Naht- und Nahtknüpftechnik. Naila: 2017. 1 p.

Zusätzlich wurden die Bestellkataloge der Firmen ausgewertet.
